# Building Pandemic-Resilient Primary Care Systems: Lessons Learned From COVID-19

**DOI:** 10.2196/47667

**Published:** 2024-02-23

**Authors:** Yejin Jeong, Trevor Crowell, Anna Devon-Sand, Theadora Sakata, Amelia Sattler, Shreya Shah, Timothy Tsai, Steven Lin

**Affiliations:** 1 Division of Primary Care and Population Health Department of Medicine Stanford University School of Medicine Redwood City, CA United States

**Keywords:** barrier, COVID-19, digital health, implementation, internet-based care, pandemic, primary care, resilience, resilient system, telehealth, telemedicine

## Abstract

On January 30, 2023, the Biden Administration announced its intention to end the existing COVID-19 public health emergency declaration. The transition to a “postpandemic” landscape presents a unique opportunity to sustain and strengthen pandemic-era changes in care delivery. With this in mind, we present 3 critical lessons learned from a primary care perspective during the COVID-19 pandemic. First, clinical workflows must support both in-person and internet-based care delivery. Second, the integration of asynchronous care delivery is critical. Third, planning for the future means planning for everyone, including those with potentially limited access to health care due to barriers in technology and communication. While these lessons are neither unique to primary care settings nor all-encompassing, they establish a grounded foundation on which to construct higher-quality, more resilient, and more equitable health systems.

## Overview

Alongside testing and vaccinations, telehealth was the third leg of the 3-legged stool representing the United States’ primary care strategy against COVID-19. Telehealth use surged early in the pandemic as patients and providers sought ways to safely access and deliver care. Improved attitudes toward telehealth, coupled with regulatory changes to reimbursements, enabled investments in internet-based care and digital health to skyrocket [1,2]. With the announcement that the United States planned to end the public health emergency for COVID-19 in May 2023 [3], patients, providers, and health systems are entering a new chapter, and a critical question remains: How will primary care systems in the United States sustain and optimize the positive changes that resulted from pandemic response efforts while continuing to address the challenges? In this paper, we describe 3 important lessons learned from the shift to internet-based care during COVID-19 that will facilitate the implementation of high-quality, resilient primary care systems.

## Lesson 1: Clinical Workflows Must Support Both In-Person and Internet-Based Care Delivery

At the onset of the COVID-19 pandemic, public health mandates abruptly shifted the basic structure of how patients interfaced with the health care system at a time when access was paramount. Necessary infection prevention measures, such as shelter-in-place and social distancing, spurred a remarkable transformation in which new processes for telehealth were designed and implemented rapidly to meet emerging demands [4]. Going forward, clinical workflows must support both in-person and internet-based care delivery, with special attention paid to ensuring consistent preventive care and chronic disease management across all care contexts. For example, traditional in-clinic rooming procedures such as preventive care reminders could be conducted asynchronously through electronic previsit questionnaires or phone calls.

Accounting for a broad spectrum of care delivery models when designing clinical workflows provides flexibility, allowing health systems to be nimble and provide high-quality care even when faced with unanticipated constraints. To accomplish this, systems must outline the essential components of care delivery and explore how to make each component accessible, taking into consideration patient factors. Systems should build pathways that efficiently connect patients with the most appropriate type of encounter (eg, clinic, telehealth, group, e-visit, e-consult, home visit, or referral), hosted by the most relevant team members (eg, physician, advance practice provider, registered nurse, clinical pharmacist, behavioral health provider, social worker, case manager, or medical assistant). Systems should recognize and address tasks that limit flexibility and convenience, such as patient forms that need to be completed on paper, scanned, and faxed. Resilient systems will use structured quality improvement methodologies to identify opportunities for innovation and the implementation of new workflows and technologies that enhance both in-person and internet-based care delivery.

## Lesson 2: The Importance of Asynchronous Care Delivery Integration Within Primary Care

Another trend that has significant implications for health care delivery in primary care is the rise in asynchronous care modalities. Methods of asynchronous care include patient portals, remote patient monitoring (RPM), and mobile health (mHealth) apps. Patient portals enable patients to view and schedule appointments, view medical test results, view and request medications, and communicate with their providers through a secure platform [5]. Asynchronous care may also leverage patient-generated health data (PGHD), examples of which include health history, biometric data, and patient-reported outcome measures. PGHD can be transmitted through mHealth apps and connected health devices, including blood pressure cuffs, glucometers, and fitness trackers that use an application programming interface (API), which allows information to be sent back and forth between an app and the primary care team. Some of our most costly chronic diseases, such as hypertension, diabetes, and behavioral health, can benefit from an increased number of telehealth visits with the health care system [6]. RPM applications can be the platform that provides frequent interactions, improved access to care, and improved health equity [7].

Digital health tools such as RPM and health apps are changing how patients interact with primary care. Patients are relying more on their smartphones and devices for prevention, diagnosis, and disease management. This provides a significant opportunity, as these tools can lead to improved quality of care and cost savings [8]. Specific examples in primary care include improving hemoglobin A_1c_ control in patients with diabetes and improving blood pressure control in patients with hypertension [9]. RPM care management and transitional care programs for congestive heart failure can reduce hospitalization days and death [10]. A multidisciplinary team-based approach is an important part of a successful RPM program, with a shift from the role of a single provider-patient relationship to a care team–patient relationship. Care team members can include primary care physicians, advanced practice providers, registered nurses, clinical pharmacists, behavioral health providers, social workers, case managers, or medical assistants. For example, with hypertension, the comparative effectiveness of 8 implementation strategies found that team-based care models including a nonphysician team member who titrates medications are the most effective for blood pressure control [11]. As the volume of available tools continues to rise, it is increasingly important for care teams to determine which technologies to recommend for their patients [12]. Textbox 1 outlines these clinical, usability, and technical considerations.

**Key considerations for integration of asynchronous digital health tools within primary care.**

**Clinical**

- Does the tool align with evidence-based medicine?
- Does the tool improve clinical care?
- Is the tool safe?
- How will the tool be integrated in the clinical workflow?
- Are there protocols for urgent issues or out-of-range remote patient monitoring measurements?
- Is there evidence of closed-loop communication with the patient?

**Usability**

- Are there training and education needs for patients and staff?
- Is the tool easy to use for patients and staff?
- Does the tool provide knowledgeable insights to patients and staff?
- Can the tool help mitigate increasing health care team burden and burnout?
- Are there any reimbursement considerations?

**Technical**

- What technology is used?
- What input data is required?
- If artificial intelligence is used, is it explainable?
- Is the tool accurate?
- Is the tool secure with reliable user authentication?
- Does the tool meet industry data security standards?

## Lesson 3: Planning for the Future Means Planning for Everyone

A final lesson learned during the pandemic is that while a transition to digital health platforms improved access for some, it exacerbated and created new barriers for others. Patients without a stable internet connection or appropriate hardware to support a telehealth visit faced a “tech gap” that left them without any way of seeing their medical providers if they belonged to one of the many health systems that suspended in-person visits. While the underpinnings of this gap stem from several channels, ranging from affordability to infrastructural shortcomings to global supply chain delays, addressing issues of digital health access before the next pandemic will minimize the number of patients who get left behind. Already, some health care systems, such as the US Veterans Administration, have incorporated a “digital divide consult” into their cadre of services [13]. Through this program, veterans are able to access tablets and cellular data plans at no cost. Though not every system will be able to intervene at this level, including a baseline technological assessment as part of a social history might at least alert providers to which patients will need additional outreach.

Language and communication barriers that already created inequities in health care before the pandemic played an even larger role during the pandemic [14]. As procedures for accessing care evolved rapidly, many clinics alerted patients to new scheduling and visit requirements through audio recordings, websites, and signs on clinic doors. More often than not, these notices were provided in only English, which presented difficulties for patients with limited English proficiency to access and use telehealth services. Additionally, as visits moved into the internet-based space, some providers had limited access to video platforms able to support more than 2 parties, thus barring the ability to use third-party interpretation services to communicate with non–English-speaking or hard-of-hearing patients. In order to optimize our current delivery system, we must not only be able to communicate changes to our non–English-speaking patients or patients with disabilities in real-time, but we must also ensure that interpretation is integrated into the digital platform with additional support before, during, and after their internet-based visits. As efforts are being made to address these barriers, there should be a concurrent effort to gather quality metrics to measure the impact of these efforts.

## Conclusion

While these lessons are neither unique to primary care nor all-encompassing, they generate insights into the work set before us. A combination of in-person and internet-based care, adaptable workflows with integrated asynchronous patient care, and an awareness of threats to health equity could address existing gaps and provide opportunities for building better systems. As the public health emergency for COVID-19 ends, let us not waste these valuable lessons in creating a future of primary care built to withstand future pandemics and other crises yet to come.

## Abbreviations

API: application programming interface
mHealth: mobile health
PGHD: patient-generated health data
RPM: remote patient monitoring

## Data Availability

Data sharing is not applicable to this article as no data sets were generated or analyzed during this study.
